# Disulfidptosis-related signatures for prognostic and immunotherapy reactivity evaluation in hepatocellular carcinoma

**DOI:** 10.1186/s40001-023-01535-3

**Published:** 2023-12-06

**Authors:** Jiajing Zhao, Zeminshan Luo, Ruizhi Fu, Jinghong Zhou, Shubiao Chen, Jianjie Wang, Dewang Chen, Xiaojun Xie

**Affiliations:** Department of General Surgery, the First Affiliated Hospital of Shantou University Medical College, Shantou, 515000 China

**Keywords:** Disulfidptosis, Hepatocellular carcinoma, Prognosis, Immunotherapy, Clustering

## Abstract

**Background:**

Hepatocellular carcinoma (HCC) is one of the most common cancers in the world and a nonnegligible health concern on a worldwide scale. Disulfidptosis is a novel mode of cell death, which is mainly caused by the collapse of the actin skeleton. Although many studies have demonstrated that various types of cell death are associated with cancer treatment, the relationship between disulfidptosis and HCC has not been elucidated.

**Methods:**

Here, we mainly applied bioinformatics methods to construct a disulfidptosis related risk model in HCC patients. Specifically, transcriptome data and clinical information were downloaded from the Gene Expression Omnibus (GEO), International Cancer Genome Consortium (ICGC) and The Cancer Genome Atlas (TCGA) database. A total of 45 co-expressed genes were extracted between the disulfidptosis-related genes (DRGs) and the differential expression genes (DEGs) of liver hepatocellular carcinoma (LIHC) in the TCGA database. The LIHC cohort was divided into two subgroups with different prognosis by k-mean consensus clustering and functional enrichment analysis was performed. Subsequently, three hub genes (CDCA8, SPP2 and RDH16) were screened by Cox regression and LASSO regression analysis. In addition, a risk signature was constructed and the HCC cohort was divided into high risk score and low risk score subgroups to compare the prognosis, clinical features and immune landscape between the two subgroups. Finally, the prognostic model of independent risk factors was constructed and verified.

**Conclusions:**

High DRGs-related risk score in HCC individuals predict poor prognosis and are associated with poor immunotherapy response, which indicates that risk score assessment model can be utilized to guide clinical treatment strategy.

**Supplementary Information:**

The online version contains supplementary material available at 10.1186/s40001-023-01535-3.

## Introduction

Hepatocellular carcinoma (HCC) is one of the most prevalent malignancies with poor prognosis in the world and a significant global health care concern [1, 2]. In 2020, there were approximately 906,000 new cases of primary cancer of the liver and 830,000 deaths globally, with HCC accounting for the majority of cases [3]. Many opportunities for early intervention, such as microwave radiofrequency ablation and surgical resection, are missed due to the difficulty of making an early diagnosis of HCC [4]. Conversion therapy, which is aim to convert unresectable or potentially resectable advanced liver cancer lesions into resectable lesions, has been a key topic in the treatment of advanced HCC in recent years [5]. Atezolizumab–bevacizumab combination therapy has become first-line therapy in some Asian countries for patients who are not candidates for radical therapy or transarterial chemoembolization [6]. Despite this, the overall survival of patients with advanced HCC has only marginally improved [7]. Early intervention and detection of HCC are critical measures in determining the patient's prognosis [8]. Therefore, biomarkers for early diagnosis, risk assessment, prognostic prediction, and the development of immunotherapy reactivity models are crucial for improving the prognosis of HCC patients and guiding clinical treatment.

Cell death plays a crucial role in the development of tumors and is inextricably linked to cancer treatment [9, 10]. Based on functional differences, cell death can be classified into accidental cell death (ACD) and regulated cell death (RCD). In recent years, various RCD mechanisms have been discovered by scientists, including but not limited to cuproptosis, ferroptosis, and reticulocyte death [11]**.** Recently, a novel form of cell death has been proposed: disulfidptosis. This study has revealed that excessive intracellular disulfide stress, resulting from cystine accumulation, can lead to rapid cell death. In glucose-deficient cancer cells with high SLC7A11 expression, the accumulation of disulfide material disrupts the normal bonding of disulfide bridges between cytoskeletal proteins, resulting in the collapse of the histone scaffold and cell death [12]. The elucidation of this cell death mechanism will aid in further understanding of cellular homeostasis and may provide novel avenue for treatment of human malignancies [13].

Therefore, we sought to further explore the association between disulfidptosis-related genes (DRGs) and HCC. At present, there are few reports on the mechanism of disulfidptosis in various cancers, and the hub genes and signaling pathways associated with disulfidptosis have not been elucidated. In our study, we applied bioinformatics approach to collect DRGs from previously published literature and extract 45 co-expressed genes with HCC-specific DEGs. K-mean clustering was performed (C1, C2). Hub genes were screened out by LASSO regression and Cox regression analysis, and then the risk score prognostic model was constructed. Additionally, we analyzed the overall survival, tumor immune, somatic mutation and clinical characteristics between high-risk score and low-risk score groups. Moreover, we developed a hub gene-related prognostic prediction model and used Tumor Immune Dysfunction and Exclusion (TIDE) database to predict the response to immunotherapy in different risk score subgroups in HCC. Ultimately, we estimated the prospective treatment agent for HCC patients with high risk scores based on the "pRRophetic" package in R.

Taken together, our risk assessment model offers novel concepts for the clinical evaluation of HCC and important information and help for the clinical choice of the appropriate treatment.

## Materials and methods

### Data collection and preprocessing

**Fig. 1:**
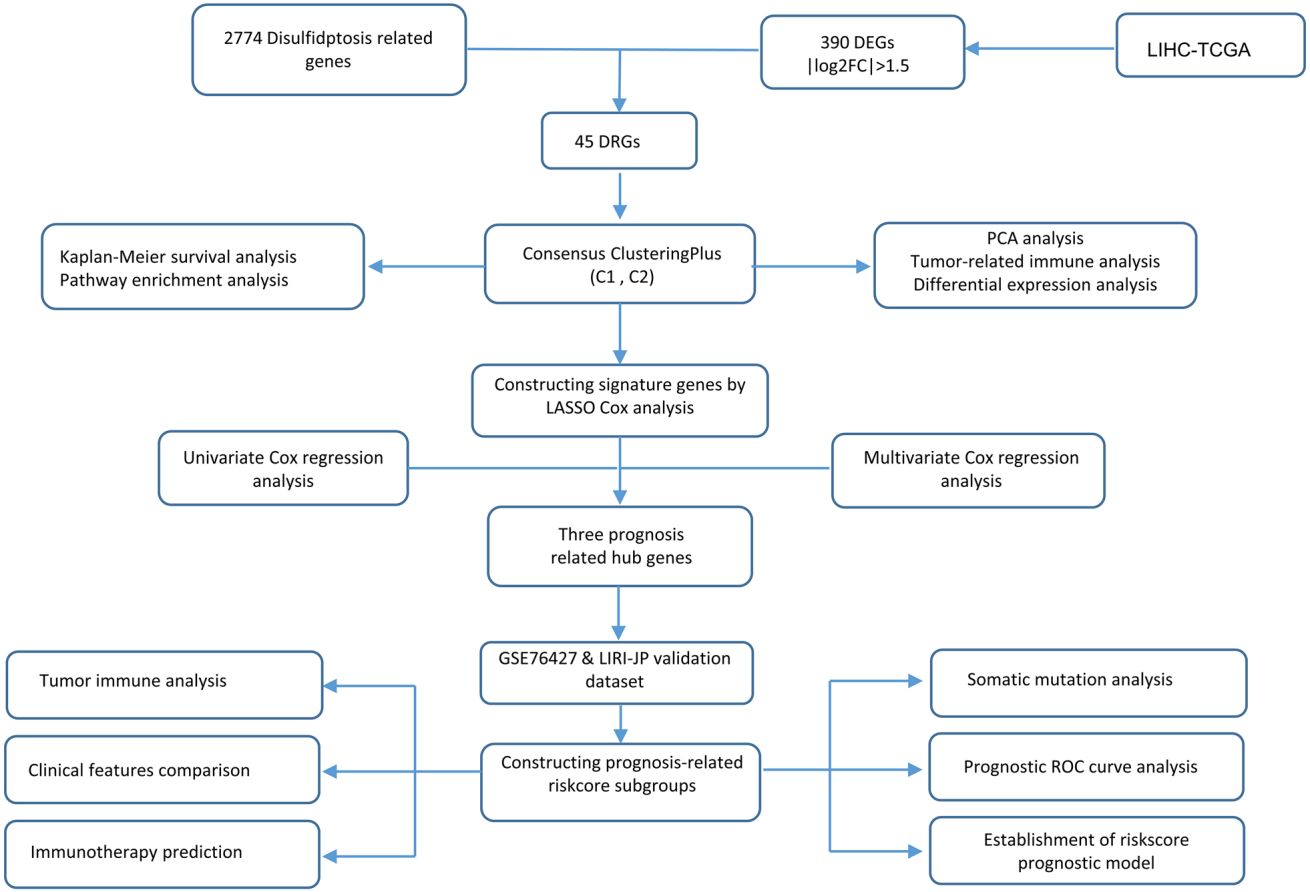
A simplified flow chart of the study

The flow diagram in Fig. 1 depicts the data collection and analysis procedure. RNA-seq, somatic mutation data, and clinical information data (TCGA-LIHC) were downloaded from The Cancer Genome Atlas (TCGA) database, which included in total 373 tumor tissue samples and 51 para-carcinoma tissue. Data lacking complete clinical information or with zero overall survival were excluded. The data from Level 3 HTSeq-FPKM are translated to transcripts per million reads (TMP). Additionally, a total of 358 HCC samples were collected from the GEO (ID: GSE76427) and ICGC-HCC (LIRI-JP) were considered as the validation set of this study. Notably, patients with incomplete clinical information or short overall survival time (< 60 days) were excluded. The complete clinical baseline data are summarized in Additional file 2. The data were subjected to the same processing. Gene Set Enrichment Analysis (GSEA) pathway data were obtained from MSigDB Collections (c2.cp.v7.2.symbols.gmt [Curated]). The false discovery rate (FDR) of 0.25 and the p < 0.05 were used as enrichment cut-offs. “ClusterProfiler” and “ggplot2” packages were applied to GSEA analysis and visualization of different cluster subgroups, respectively. Furthermore, hub genes promoter methylation and total protein data were downloaded from UALCAN database, and immunohistochemical data were obtained from The Human Protein Atlas (HPA) database.

### Extraction of disulfidptosis-related genes

As the DRGs of this research, we extracted 2,774 genes with a norm Z > 1 of the relative change ratio of CRISPR-Cas9 screening of SCL7A11-high 786-O cells under conditions of glucose replete and hunger from the previously published literature [12]. The complete gene list is summarized in Additional file 2.

### Differentially expressed genes in HCC

“limma” package was utilized for the analysis of DEGs in tumor and non-tumor tissues in HCC. Additionally, “ggplot2” and “ComplexHeatmap” packages were applied for visualization of DEGs. Then, Venn diagram was generated to visualize the 45 co-expressed genes. Furthermore, “igraph” and “ggraph” packages were powered to generate the network of 45 co-expressed genes.

### Consensus clustering

Based on the above 45 co-expressed genes, 373 patients from the TCGA-LIHC cohort were used for consensus clustering using the k-mean clustering algorithm to obtain two different clustering subgroups (C1, C2). Moreover, principal component analysis (PCA) was utilized to determine the separation of two cluster subgroups. In addition, heatmap and Kaplan–Meier (KM) curve were generated to visually compare 45 co-expressed genes as well as overall survival between the two clustering subgroups.

### Enrichment analysis of genes and signaling pathways

The DEGs of the two clustering subgroups were analyzed by “limma” package, and then the GO and KEGG gene enrichment analysis were conducted. Bubble plots were generated for visualization of GO and KEGG analysis’ results. In addition, GSEA analysis was powered to study DEGs-related signaling pathways between C1 and C2 subgroups.

### Construction and identification of prognostic model

The LIHC cohort's prognosis-related genes were extracted through LASSO Cox regression analysis. In addition, univariate and multivariate Cox regression were utilized to further extract core genes, resulting in the identification of three hub genes (CDCA8, SPP2 and RDH16) that were considerably associated with prognosis. In addition, risk score was calculated for each HCC patient, and the calculation formula was risk score = ∑Coef_i_ x Exp_i_. The LIHC training cohort was divided into high risk score and low risk score subgroups according to the median of risk score. KM survival analyses were performed for two different subgroups and receiver operating curves (ROC) were established to assess the 1-, 3-, 5-year reliability of the model predictions.

### Clinical characteristics and prognosis of risk score-related subgroups

The Wilcoxon rank sum test was utilized to assess the variances in risk score between patients with distinct clinical stages (TMN) and grades. In addition, KM survival curves were used to examine the overall survival of HCC patients with various clinical characteristics, and the log-rank approach was employed to calculate the statistical difference between high risk score and low risk score subgroups.

### Evaluation of tumor-associated immune microenvironment and drug reactivity

The Microenvironment Cell population-counter (MCP-counter) method was powered to evaluate the infiltration of immune cells in HCC tumor tissues. The bar chart was generated to compare the enrichment degree of immune cells in 10 tumors immune microenvironment among different risk score subgroups. Meanwhile, we further observed the expression differences of immune checkpoint inhibitors and HLA-related genes. Scatter plots were powered to determine the correlation between the risk score and the level of tumor immune cell infiltration. Moreover, we also used the ESTIMATE algorithm to evaluate the somatic score, immune score and ESTIMATE score of the LIHC samples. Furthermore, TIDE database was utilized to predict the responsiveness of HCC patients to immunotherapy. Lastly, we predicted the prospective treatment drug for high-risk HCC patients using the R package "pRRophetic".

### Establishment of predictive models for prognostic risk factors

Based on univariate and multivariate Cox regression analysis, a prognostic nomogram of risk factors was constructed. Decision curve analysis (DCA) and calibration curve were generated to assess the accuracy of the prognostic nomogram.

### Statistics

All data in this study were implemented in R (v.4.2.1). Spearman method is powered to determine the correlation between two variables. Log-rank method was utilized to compare the difference in overall survival between the two subgroups. The Wilcoxon rank sum test was applied to identify inter-group differences between different variables. In all statistical methods, *p* < 0.05 was regarded as a significant statistical difference.

## Results

### Extraction of DEGs and DRGs co-expressed genes in HCC

**Fig. 2:**
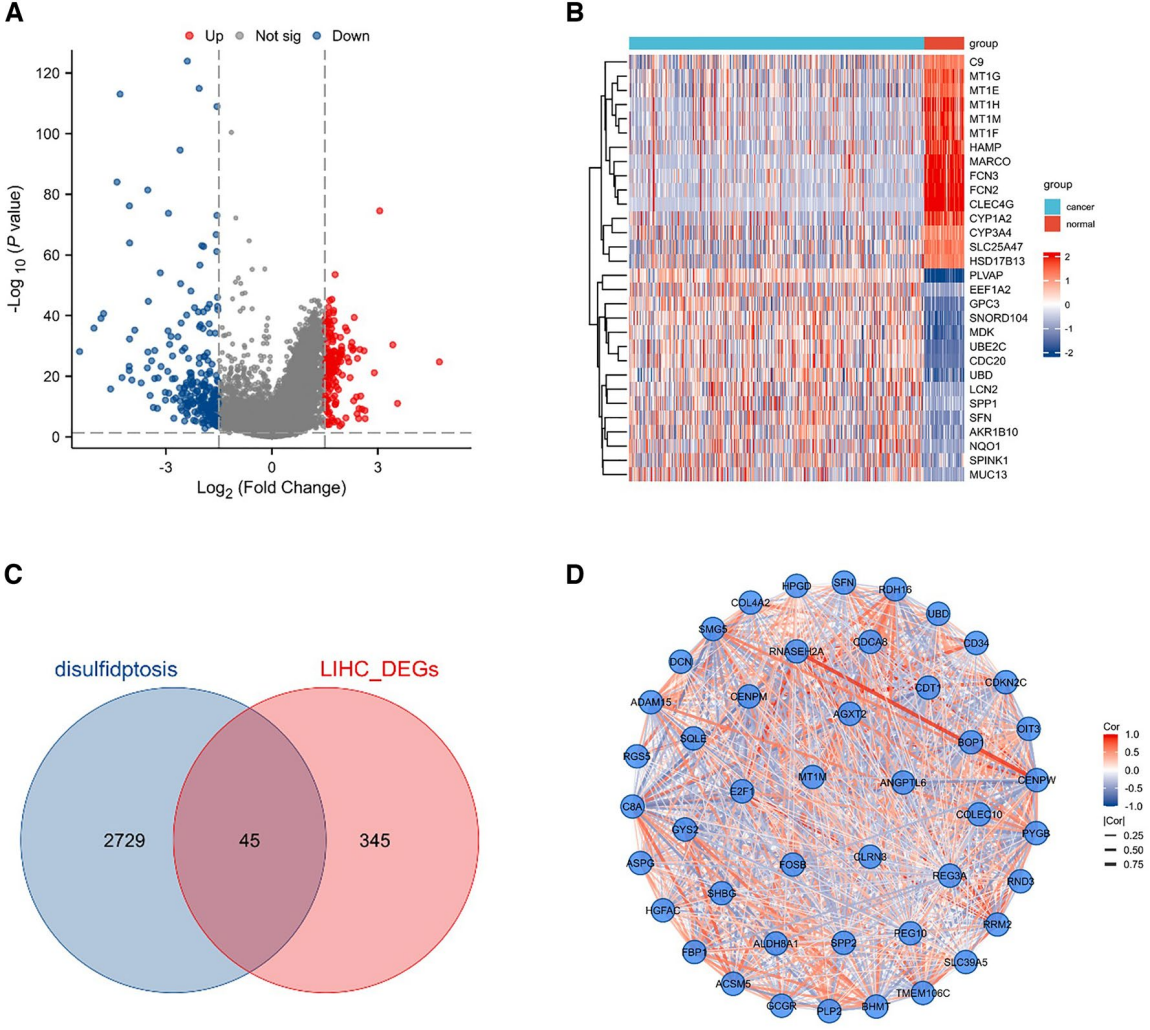
Identification of co-expressed genes. **A** Volcanic plot of DEGs in tumor and normal tissues of HCC patients. **B** Heatmap of 30 top representative DEGs in HCC patients. **C** A Venn diagram is generated to represent 45 co-expressed genes of DRGs and DEGs. **D** Protein interaction network diagram of 45 co-expressed genes

To understand the DRGs set associated with HCC, “limma” method was utilized to identify DEGs between normal tissues and tumor tissues. | log2FC |> 1.5 was considered as significantly differentially expressed genes. Finally, we get 229 downregulated genes and 161 upregulated genes. Volcano maps and heat maps were generated to show the final results **(**Fig. 2A, B). In addition, our previously collected 2774 DRGs were intersected with the above 390 DEGs, resulting in 45 co-expressed genes. Venn diagram was used to visualize the 45 co-expressed genes **(**Fig. 2C**)**. To further understand the interconnections between the 45 genes, the spherical network diagram was generated to display the correlation between each gene **(**Fig. 2D**)**.

### Consensus clustering associated with 45 co-expressed genes

**Fig. 3:**
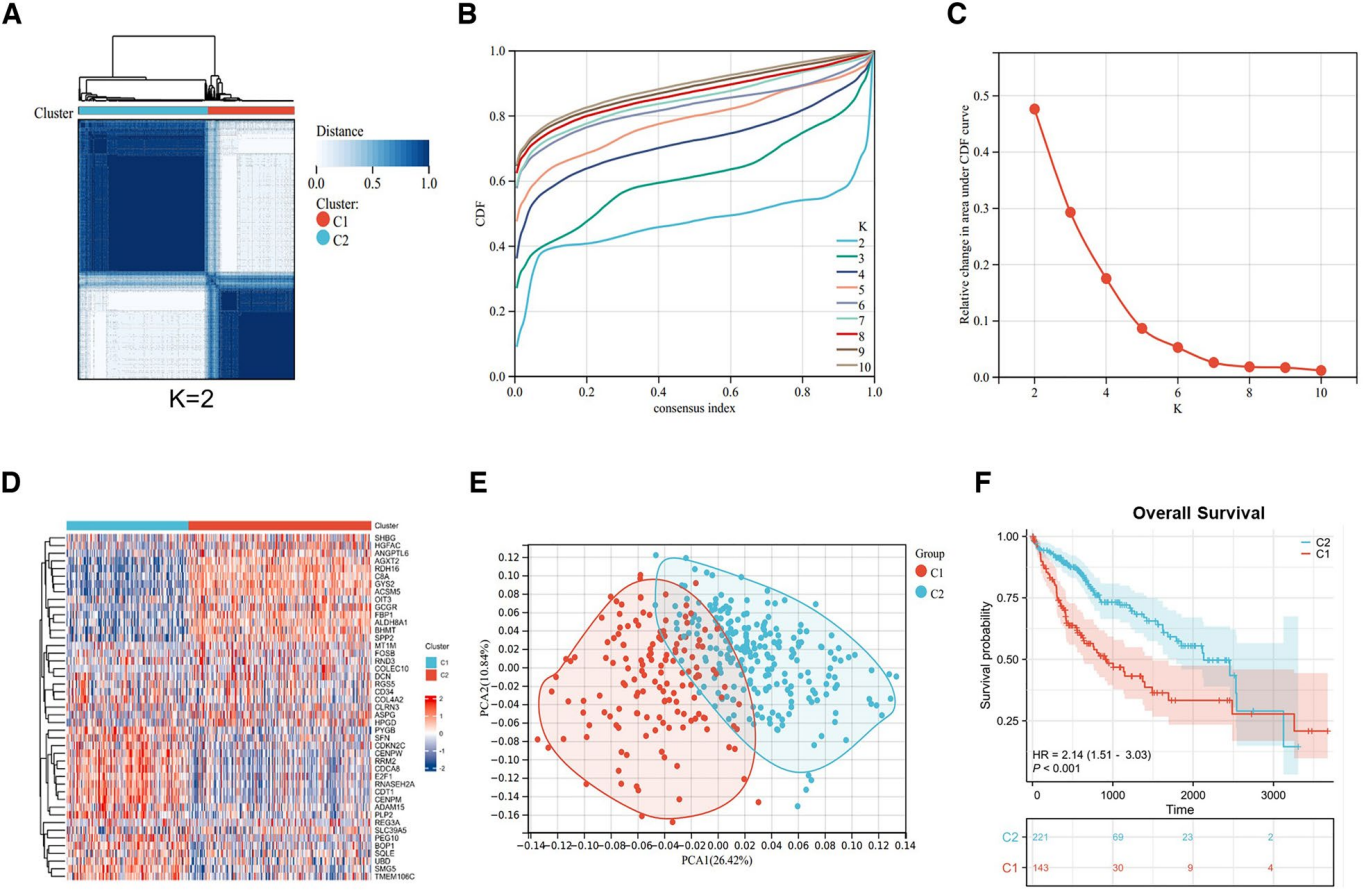
Consensus clustering based on 45 co-expressed genes. **A** Clustering heatmap of co-expressed genes. **B** Cumulative distribution function area of different clustering (*k* = 2–10). **C** The relative change of area under the CDF curve. **D** An heatmap of 45 co-expressed genes in different clustering groups**. E** PCA plot of two clustering subgroups. **F** Analysis of overall survival between different cluster subgroups

Aim to explore the relationship between co-expressed genes and HCC subtypes, we applied k-mean algorithm to divide HCC patients into two separate clusters (C1 and C2) **(**Fig. 3A**)**. The cumulative distribution function (CDF) and the relative change under the CDF curve suggested that k = 2 was the best clustering result **(**Fig. 3B, C). Additionally, heat maps that display the expression of 45 co-expressed genes in the two subgroups were created. Results indicate that some genes, such as *ANGPTL6, AGXT2, RDH16, C8A, GYS2, ACSM5, FBP1, ALDH8A1, BHMT* and *SPP2* are highly expressed in C2. The expression of some genes, such as *SFN, CDKN2C, CENPW, RRM2, CDCA8, E2F1, RNASEH2A, CDT1, CENPM, ADAM15*, and others, is noticeably higher in C1 cluster **(**Fig. 3D**)**. To further verify the mutual independence between the two clusters, PCA was powered to verify the two subgroups. The results suggested significant separation between the two subgroups **(**Fig. 3E**)**. Moreover, C1 subgroup indicated poor prognosis according to the prognosis curve of overall survival in Fig. 3F**.**

### Enrichment of biological functions and signaling pathways

**Fig. 4:**
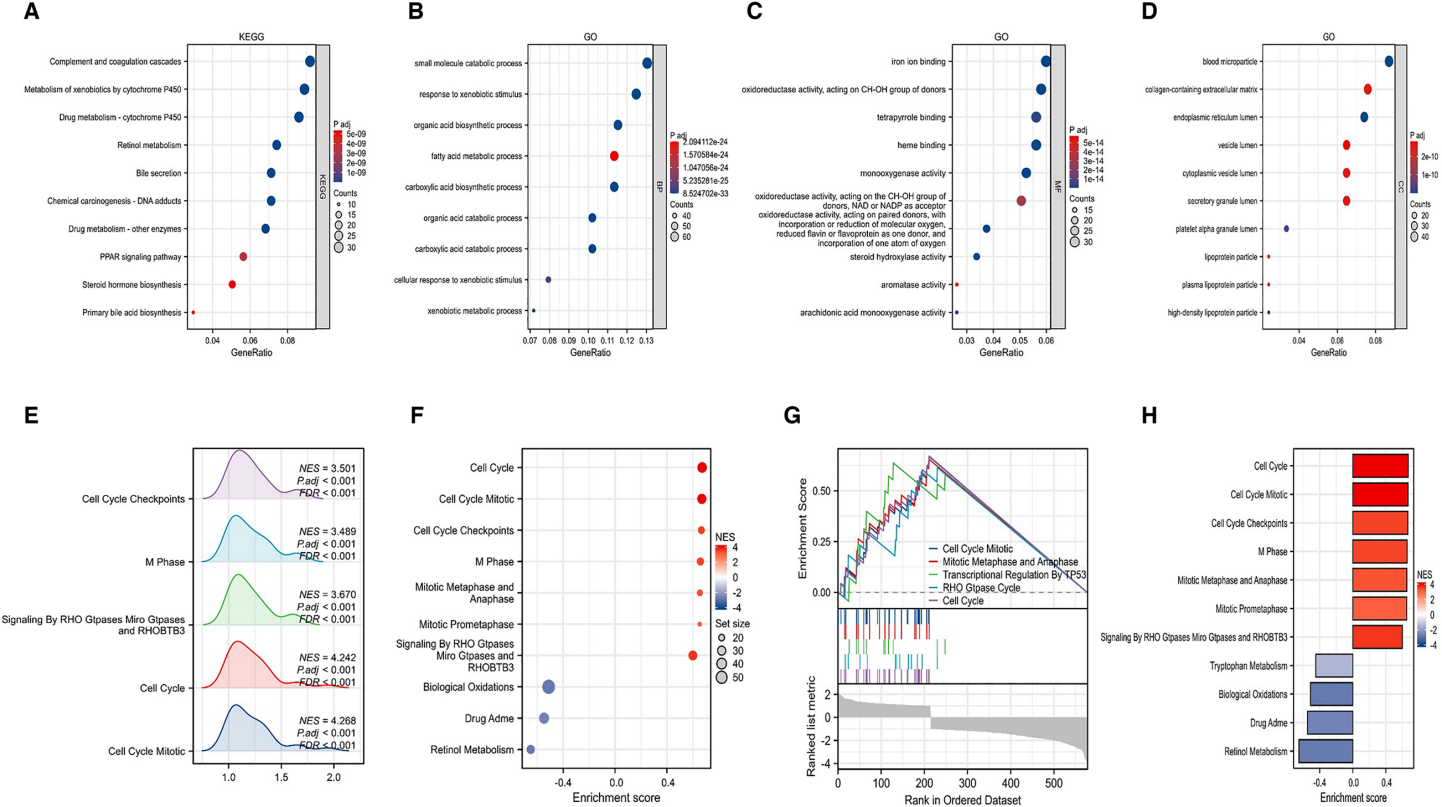
Signaling pathways and functional enrichment analysis between different subgroups. **A**–**D** Representative bubble plot analyzed by KEGG and GO. Bubble plots representing KEGG (**A**), BP**(B)**, MF (**C**) and CC (**D**) are generated, respectively. **E**, **H** Visualization of representative results analyzed by GSEA. Visualization of mountain plot (**E**), bubble plot (**F**), classic plot (**G**) and bar plot (**H**) for GSEA analysis results

To confirm whether consensus clustering result related to disulfidptosis, “limma” method was measured to identified DEGs between two subgroups, then the DEGs with | log2FC |> 1 were used for GO and KEGG enrichment analysis. Bubble maps were generated to visualize signaling pathways, biological processes, cell composition, and molecular functions, respectively **(**Fig. 4A–D). From the results, different cluster subgroups were observed to be involved in complement and coagulation system, metabolism of cytochrome P450, drug metabolism, bile secretion and retinol metabolism, PPAR signaling pathway, chemical carcinogens–DNA adducts. Meanwhile, the cellular response to xenobiotic stimulation, iron ion binding, biological oxidative stress and lipoprotein particles, endoplasmic reticulum lumen, and granular lumen were also involved in two different subgroups. Complete GO and KEGG analysis are summarized in Additional file 3. To further investigate the relevant signaling pathways of DEGs, we performed GSEA enrichment analysis of them. The results showed that the DEGs between two cluster subgroups were mainly involved in cell cycle and metaphase, oxidative stress and retinol metabolism. Mountain map, bubble plot, GSEA classic plot and bar chart were generated, respectively, for comprehensive visualization of GSEA enrichment analysis results **(**Fig. 4E–H). The full GSEA enrichment results are presented in Additional file 4.

### Establishment of prognostic risk factor model

**Fig. 5:**
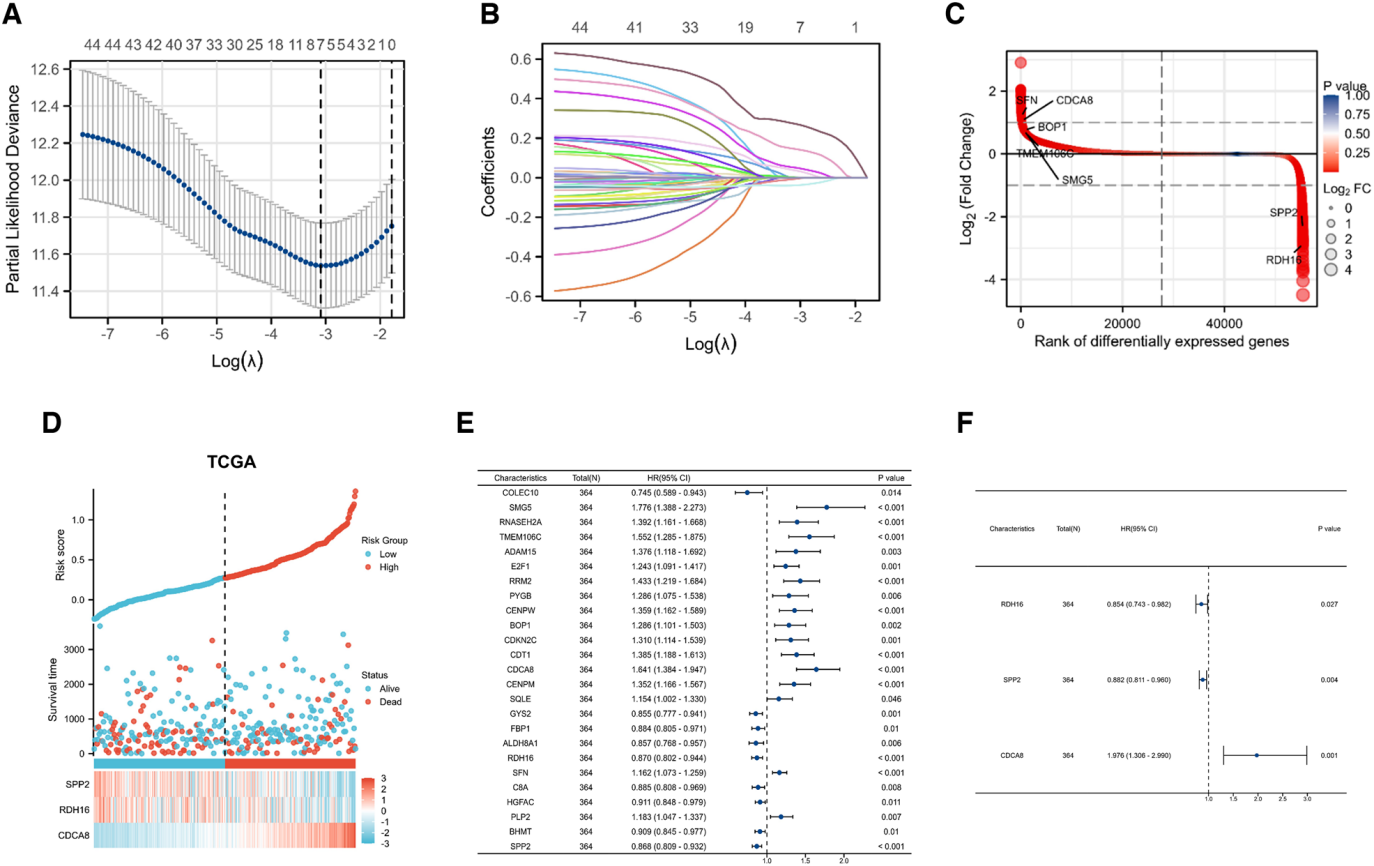
Establishment of prognostic risk factor model. **A**, **B**) Partial likelihood deviation plot and coefficient curve of LASSO Cox analysis. **C** A DEGs sequenced heatmap of 7 genes extracted by LASSO Cox regression analysis. **D** A heatmap of prognosis-related risk factors and risk score model. **E–F** Forest plots of univariate (**E**) and multivariate Cox regression (**F**)

In order to further screen genes related to prognosis, we used LASSO Cox regression analysis for extraction. Seven candidate genes that met the minimum lambda value of 0.045532 were screened out. Partial likelihood deviance plots and coefficients distribution curves were generated to visualize the LASSO Cox regression results (Fig. 5A, B). A volcano plot was utilized to show the location of the seven candidate genes in the differential gene ranking **(**Fig. 5C**)**. In order to further ensure the reliability of core genes, univariate and multivariate Cox regression analysis were used to further screen three hub genes (*RDH16*, *SPP2* and *CDCA8*). The forest plot is shown in Fig. 5E, F. Heatmap of three hub genes related to prognosis is shown in Fig. 5D.

### Risk score correlates with somatic mutations and overall survival

**Fig. 6:**
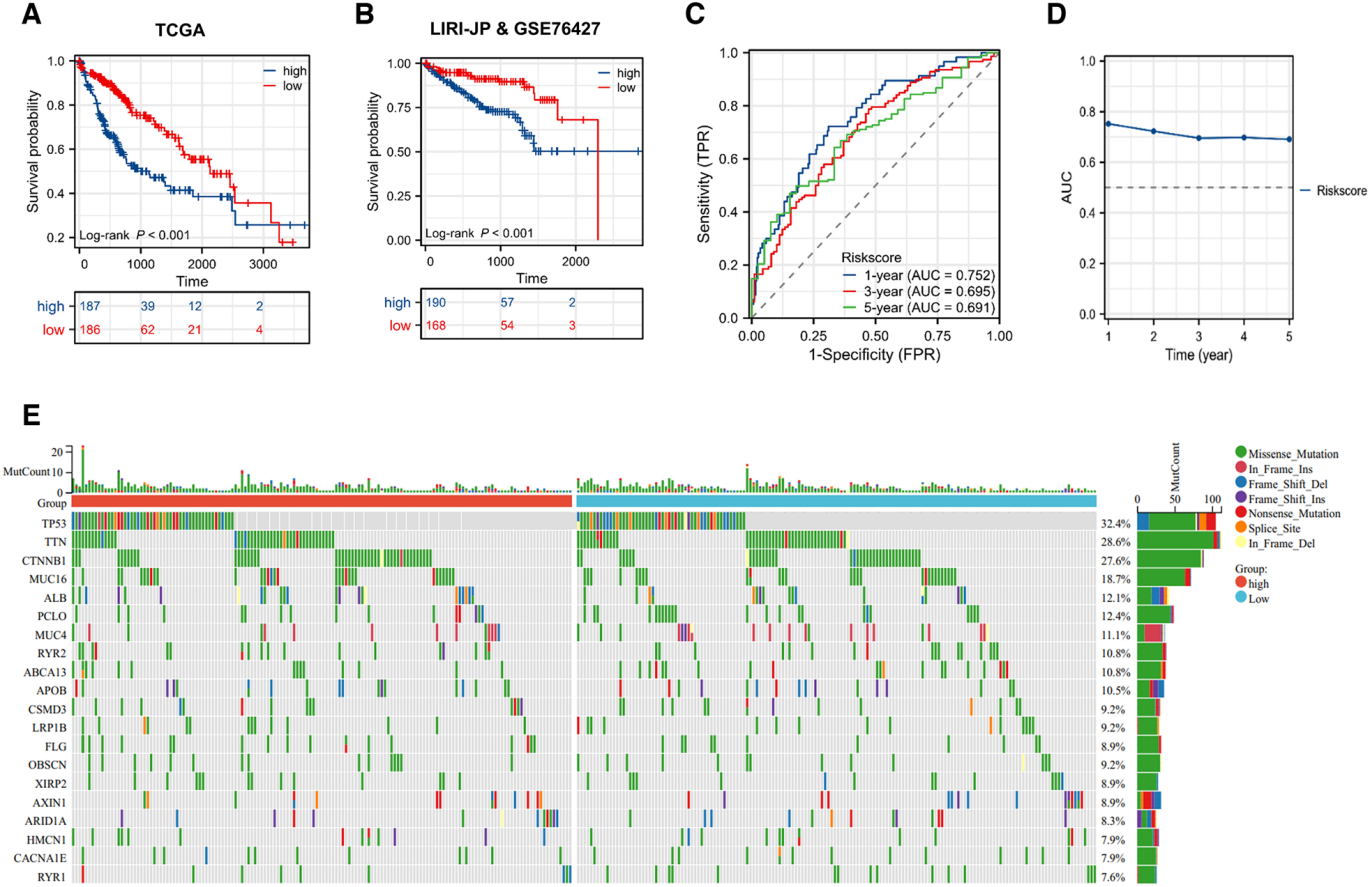
Somatic mutations and prognosis in different risk score subgroups. **A**, **B** KM curves of overall survival of different risk score subgroups in TCGA training set and LIRI-JP & GSE76427 verification set. **C**, **D** ROC curve (**C**) and broken line plot (**D**) of risk score related 1-, 3-, and 5-year overall survival prediction. **E** Waterfall plots of somatic mutations in different risk score subgroups

To investigate the relationship between risk score and prognosis, we divided the cohort into high and low risk score subgroups based on the median risk score. KM curves were generated to compare the differences in overall survival between the two risk score subgroups. The results suggested that high risk score predicted adverse prognosis (Fig. 6A). Additionally, we found similar results in the LIRI-JP&GSE76427 verification set and there was statistically significant difference (*P* < 0.001) (Fig. 6B). To further confirm the accuracy of the risk assessment model, we used ROC curve and line graph to evaluate the reliability of the risk prognosis model. The results indicate that the AUC values of 1-, 3-, and 5-year predictions of this model are, respectively, 0.752, 0.695, and 0.691, indicating that this model has a certain reliability (Fig. 6C, D). In order to observe the somatic mutation difference between the high risk score subgroup and the low risk score group, mutation data of the TCGA-LIHC training set were collected and a waterfall diagram was generated (Fig. 6E). The results showed that missense mutation accounts for the majority. In addition, *TP53* (32.4%), *TTN* (28.6%) and *CTNNB1* (27.6%) were the genes with the top three largest number of mutations in all somatic mutation.

### Risk score is correlated with clinical characteristics

**Fig. 7:**
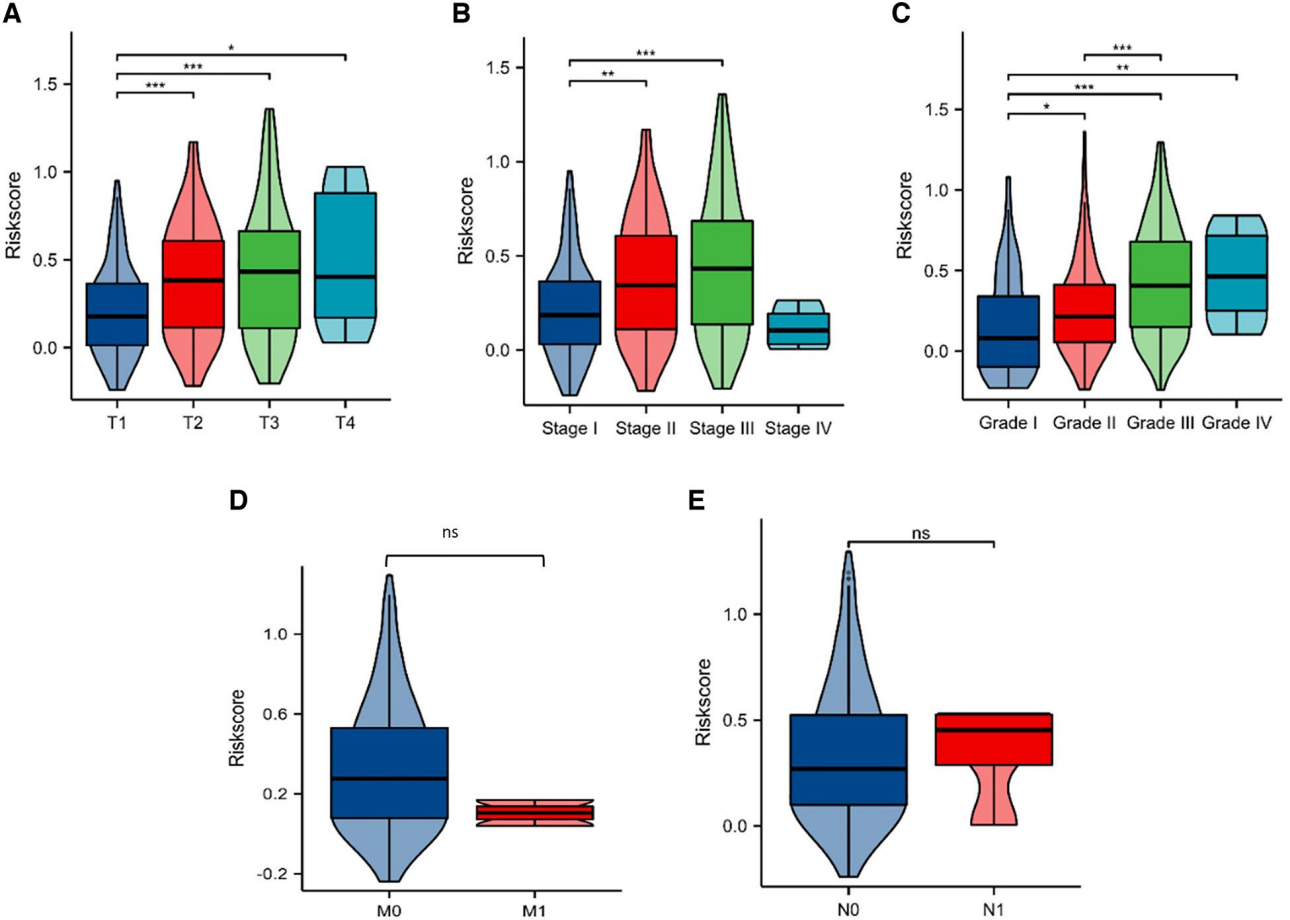
Risk score is related to the clinical characteristics of HCC patients. **A–E** Violins of risks core and tumor size (**A**), clinical stage (**B**), clinical grade (**C**), distant metastasis (**D**), and lymph node metastasis (**E**)

**Fig. 8:**
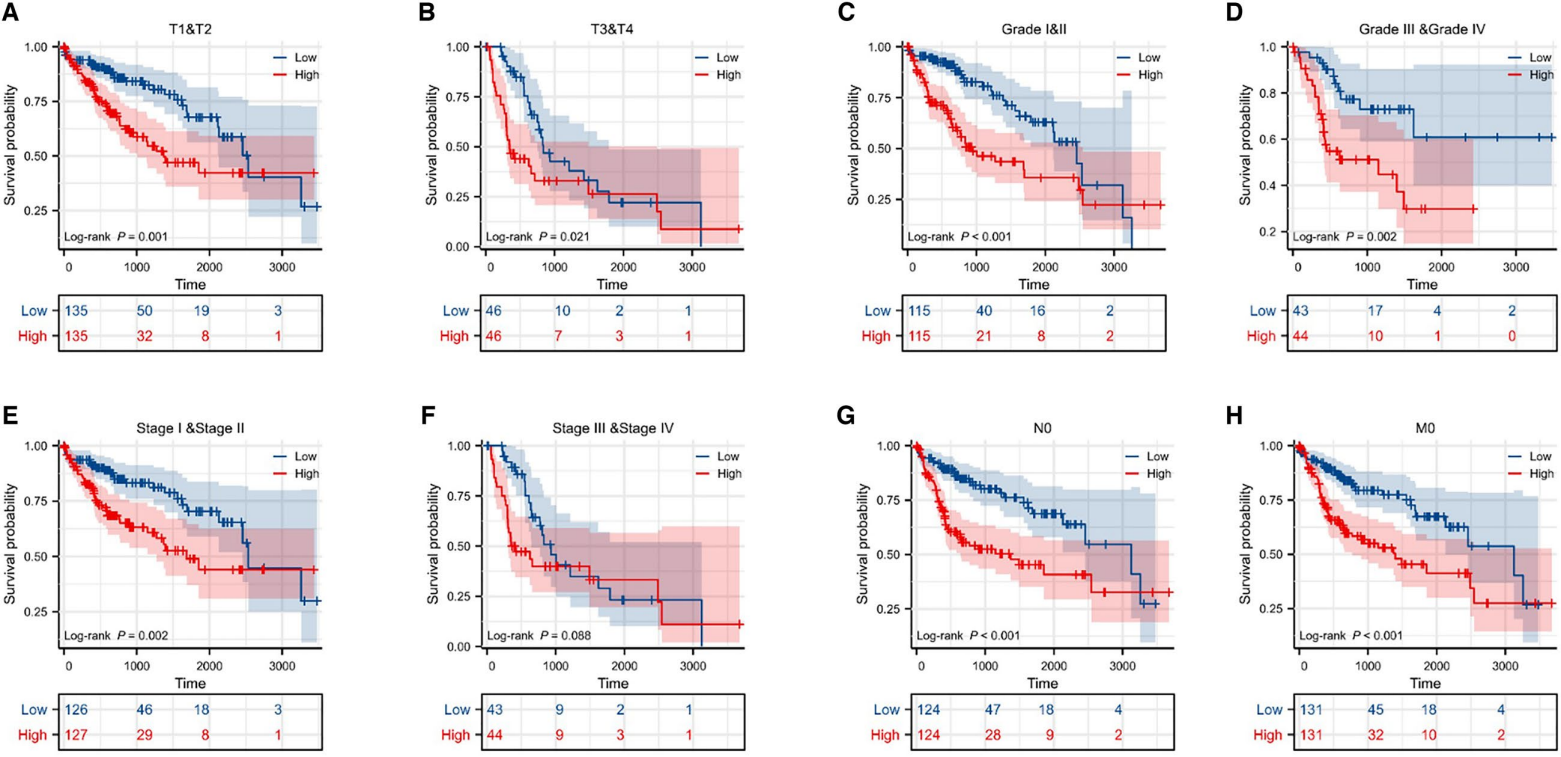
Risk score is associated with the prognosis of HCC patients with different clinical characteristics. **A**, **B** KM curves of different tumor size in different risk score subgroups. **C**, **D** KM curves of different clinical grades in different risk score subgroups. **E**, **F** KM curves of different clinical stages in different risk score subgroups. **G** KM curves of HCC patients without lymph node metastasis in different risk score subgroups. **H** KM curves of HCC patients without distant metastasis in different risk score subgroups

To visualize the risk score of HCC patients with different pathological characteristics, violin plots were generated. The results showed that there was a positive correlation between tumor diameter and risk score in HCC patients (Fig. 7A). Similarly, we found that tumor stage and tumor grade were also positively correlated with risk score (Fig. 7B, C). However, there was no significant difference in risk score between patients with lymph node or distant metastasis in HCC, which may be due to the small sample size (Fig. 7D, E). In addition, we also generated the clinical prognosis of patients with different pathological features in HCC. The results suggested that the prognosis of HCC in the high risk score subgroup was worse than that in the low-subgroup group in different stages, grade and tumor volume (Fig. 8A–F). Similarly, high risk score indicated unfavorable prognosis when there was no lymph node metastasis or distant metastasis (Fig. 8G, H).

### Tumor immune analysis of different risk score subgroups

**Fig. 9:**
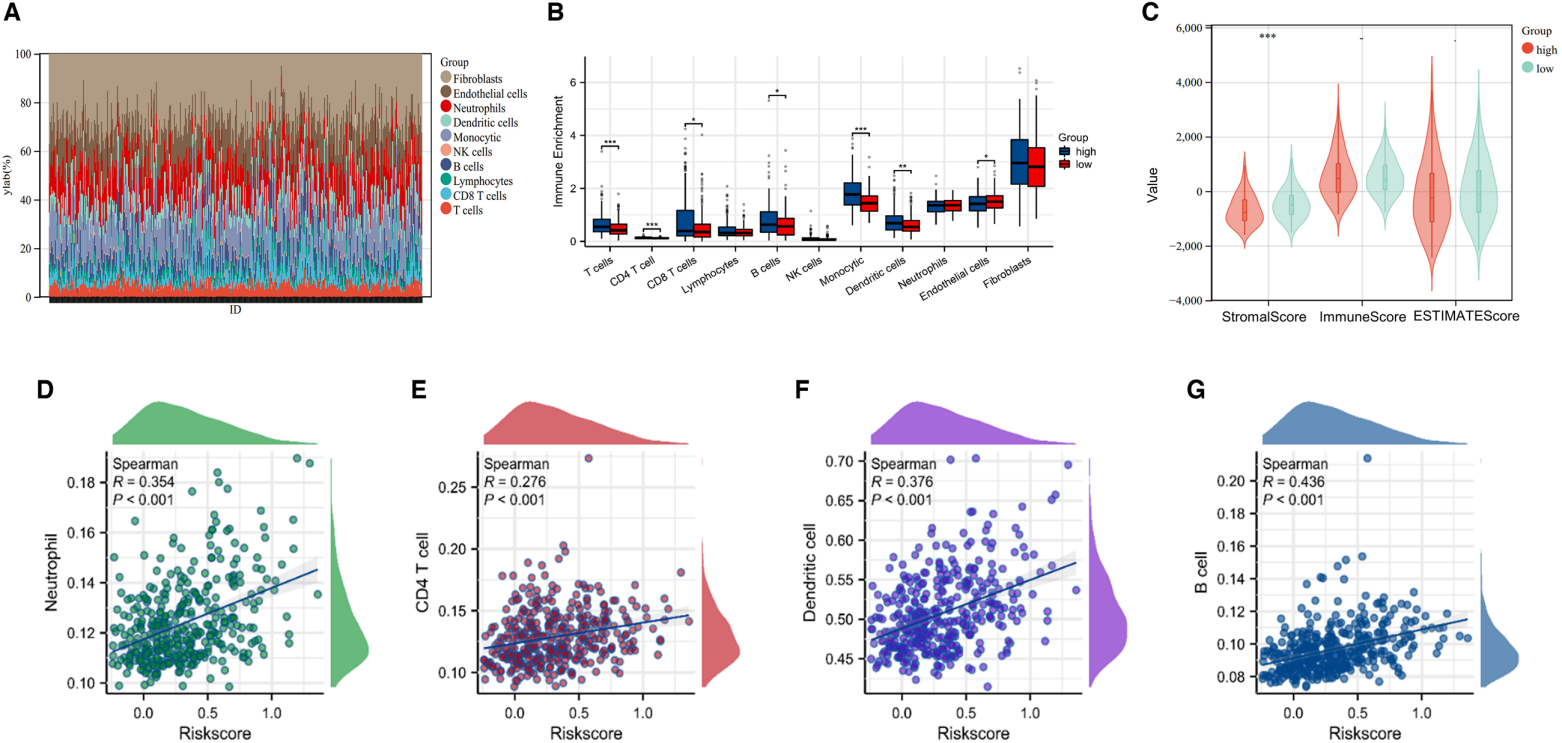
Tumor immune analysis of different risk score subgroups. **A** A stack diagram of 10 types of immune cells. **B** CMP counter score of 10 tumor-associated immune cells in different subgroups. **C** Violin plots of somatic score, immune score and ESTIMATE score between risk score subgroups. **D**–**G** Scatter plots of risk score correlation with neutrophils, CD4T cells, dendritic cells, and B cells. (*p < 0.05, **p < 0.01, ***p < 0.001, ****p < 0.0001)

To observe the infiltration levels of tumor-associated immune cells in different risk score subgroups, we used the CMP counts algorithm to calculate 10 tumor-associated immune cells. Stacked plot and bar graph were generated for visualization of the various immune cells (Fig. 9A, B). To verify the above results, we used CIBERSORT algorithm to re-evaluate the differences between different subgroups of immune cells (Additional file 1: Fig. S1). The results showed that regulatory T cells (Tregs) and CD4 + T cells were significantly enriched in the high-risk subgroup. Moreover, the ESTIMATE algorithm was applied to calculate somatic score, immune score and ESTIMATE score of two subgroups. The results showed that the stromal score of high risk score group was lower than that of low risk score group, but there was no significant difference between immunological score and ESTIMATE score (Fig. 9C). Aim to further investigate the association of immune cells with risk score, we utilized scatter plots for visualization. The results suggested that neutrophils, CD4 T cells, dendritic cells, and B cells were positively correlated with the risk score (Fig. 9D–G).

### Prediction of response to immunotherapy and chemotherapeutic drug reactivity

**Fig. 10:**
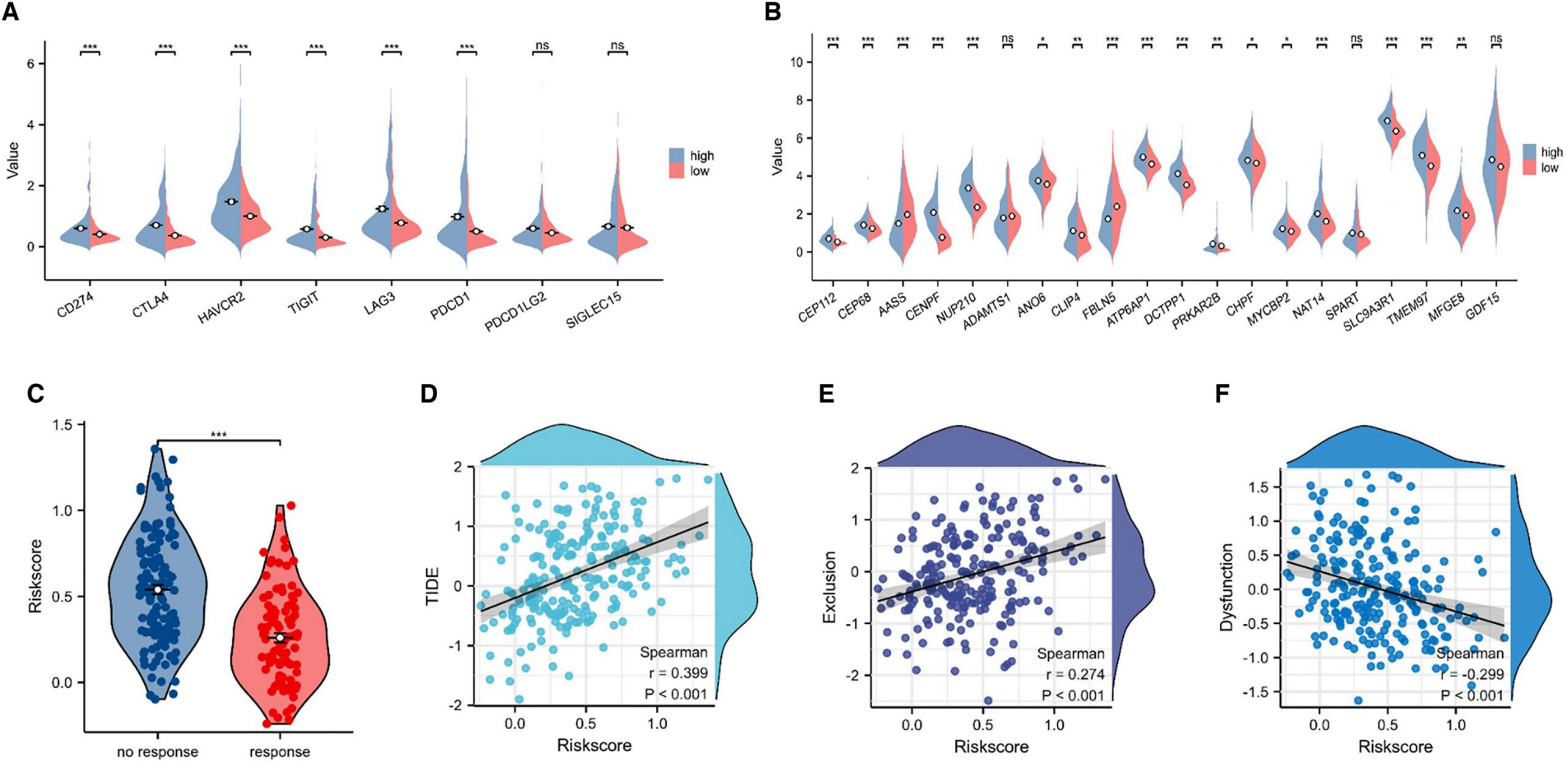
Prediction of response to immunotherapy. **A**, **B** Bar charts of ICIs-related genes (**A**) and HLA-related genes **B** in different subgroups. **C** Violin charts of risk score in HCC patients who did and did not respond to immunotherapy. **D**–**F** Correlation scatter plot of risk score with TIDE score (**D**), exclusion score **E** and dysfunction score (**F**). (*p < 0.05, **p < 0.01, ***p < 0.001, ****p < 0.0001)

To understand the responsiveness of HCC patients with different risk score subgroups to immunotherapy, we also generated violin plots to compare 8 immune checkpoint inhibitor (ICIs) genes and 20 HLA-related genes [14]. The results suggest that, most of the high risk score group had ICIs-related genes (*CDC274, CTLA4, HAVCR2, TIGIT and PDCD1*) and HLA-associated genes (*CEP112, CEP68, AASS, CENPF, NUP210, ANO6, CLIP4, FBLN5, ATP6AP1, PRKAR2B, CHPF, MYCBP2, NAT14, SLC9A3R1, TMEM97* and *MFGE8*) were upregulated, indicating that the high risk score group may have a poor response to immunotherapy (Fig. 10A, B).

**Fig. 11:**
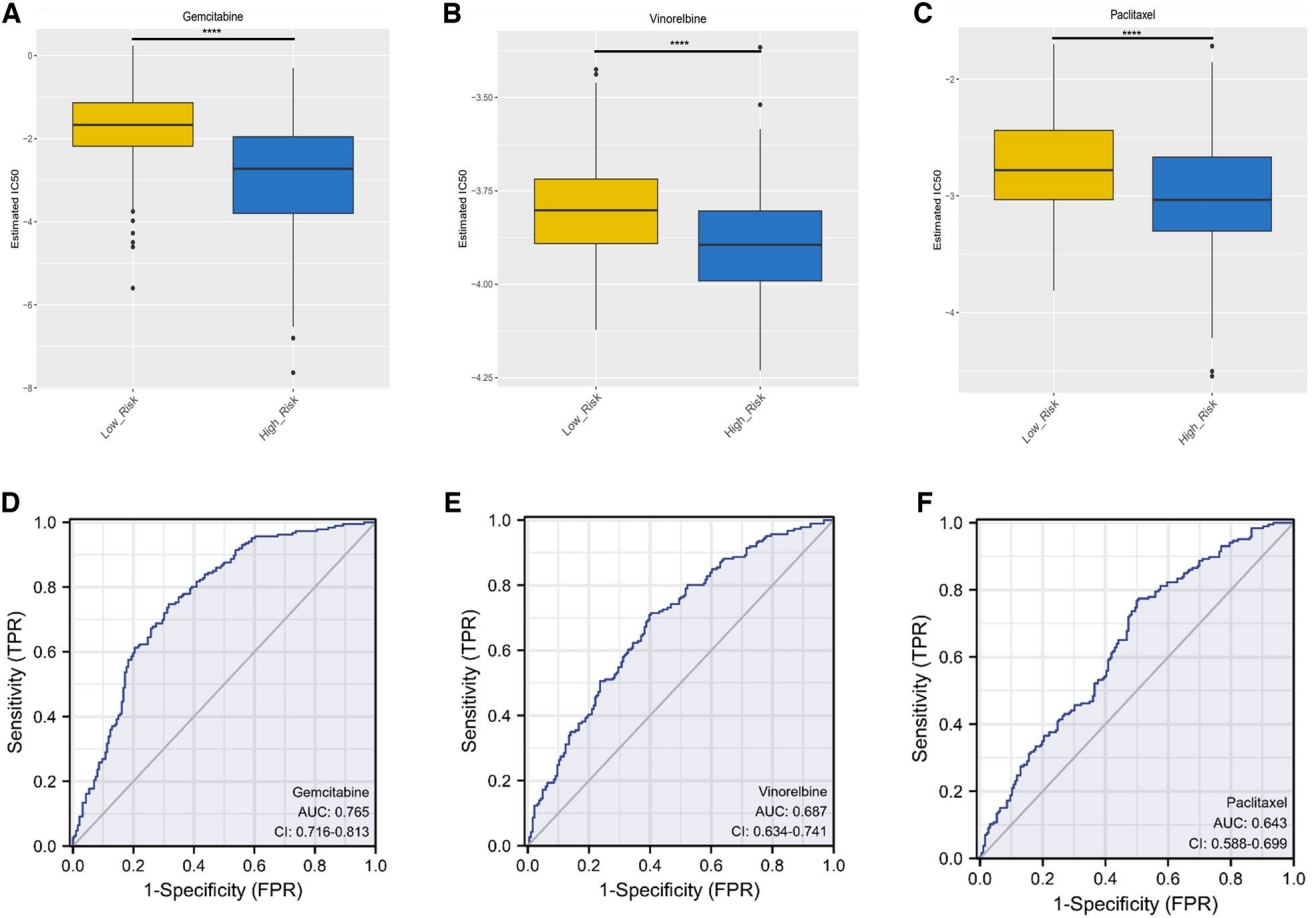
Prediction of response to chemotherapeutic drug reactivity. **A-C** IC50 bar plots of gemcitabine (**A**), vinorelbine (**B**) and paclitaxel (**C**) among different high-risk and low-risk subgroups. **D**–**F** IC50-related ROC curves of gemcitabine (**D**), vinorelbine (**E**), and paclitaxel (**F**). (****p < 0.0001)

To further explore whether the response of HCC patients to immunotherapy can be predicted by the risk score, we used the TIDE database to predict the samples of tumor patients in the TCGA-LIHC cohort. The results dedicated that the response to immunotherapy in the high risk score group is worse than low risk score group **(**Fig. 10C**)**. In addition, the result show that risk score is positively correlated with TIDE score as well as exclusion score (Fig. 10D, E), while negatively correlated with dysfunction score (Fig. 10F). "pRRophetic" package was powered to predict the half maximal inhibitory concentration (IC50) of HCC-related anti-cancer drugs. The result showed that HCC patients in the high risk score subgroup were more sensitive to gemcitabine, vinorelbine and paclitaxel (Fig. 11A–C). The ROC curve showed that the predictive value of IC50 of gemcitabine had a certain accuracy (AUC = 0.765), while the predictive value of IC50 of vinorelbine and paclitaxel was uncertain (AUC < 0.7) (Fig. 11D–F). The above results suggest that gemcitabine may be an effective therapeutic agent for the treatment of HCC in the high risk score subgroup.

### Construction of prognostic models related to risk factors

**Fig. 12:**
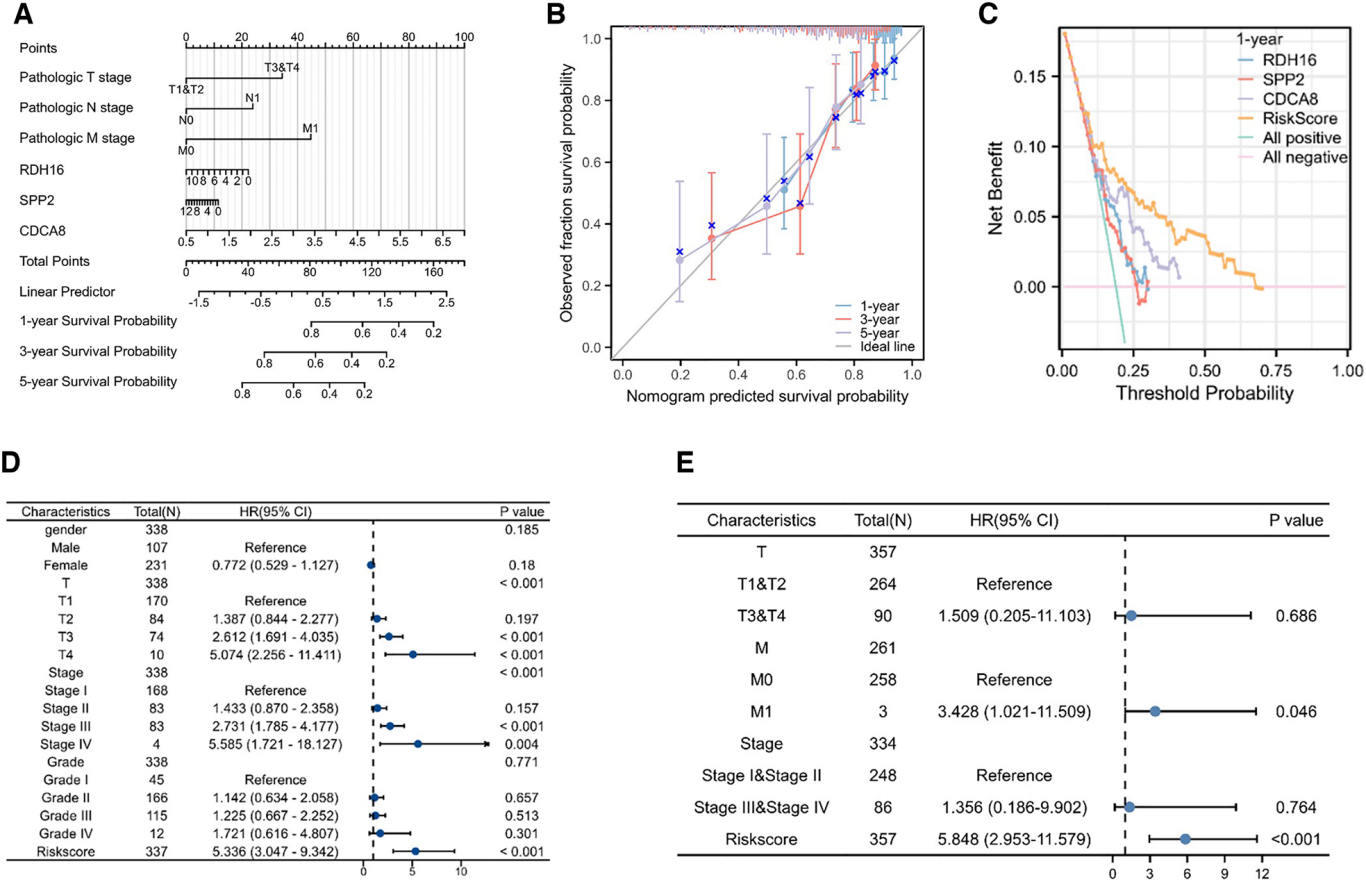
Construction of prognostic model related to risk factors. **A** A graph of independent risk factors associated with prognosis. **B**, **C** Calibration plot and DCA plot for risk score model correlation. **D**, **E** Univariate and multivariate Cox regression forest plots for prognosis-related risk factors

In order to further elaborate the relationship between independent risk factors and the prognosis of HCC patients, a nomogram was generated to construct a prognostic prediction model **(**Fig. 12A**)**. Based on the clinical characteristics and hub genes of each individual HCC patient, the overall risk score was calculated, and then the probability of survival after 1-, 3-, and 5 years was calculated. In addition, calibration plots were established to test the reliability of the prediction model **(**Fig. 12B**)**. The results showed that the 1-, 3- and 5-year survival rates predicted by the model were close to the ideal line, indicating that the prediction model was reliable. Similarly, DCA curves were generated to see the effectiveness of each independent risk factor **(**Fig. 12C**)**. Since each independent factor does not intersect the two slash lines, it indicates that the above independent factors are valid. To further determine the association of different independent risk factors with prognosis, univariate Cox regression and multivariate Cox regression analyses were used to visualize clinical characteristics. The results showed that different pathological features (TNM) and risk score were significantly associated with prognosis (Fig. 12D, E).

### Validation of hub genes at the methylation and total protein levels

To further investigate the expression of hub genes in HCC, we downloaded the immunohistochemical results of the corresponding hub genes from the Human Protein Atlas (HPA) database. In addition, the total protein expression levels of hub genes were collected in UALCAN database to validate the results. The results showed that CDCA8 was highly expressed and RDH16 was lowly expressed in HCC tumor tissues **(**Additional file 1: Fig. S2). Unfortunately, we did not find SPP2 results in the HPA database. Furthermore, we downloaded the methylation data of CDCA8, SPP2 and RDH16 in the UALCAN database. The results showed that CDCA8 (Additional file 1: Fig. S3) and SPP2 methylation levels were decreased in tumor tissues with worse clinical stages (Additional file 1: Fig. S4). There was no significant difference in the methylation level of RDH16 (Additional file 1: Fig. S5).

## Discussion

Hepatocellular carcinoma, accounting for about 75–85% of primary liver cancer, has a high incidence, rapid progression and high mortality. Traditional treatment includes radiotherapy, chemotherapy, surgery and transcatheter arterial chemoembolization (TCEA). For those advanced HCC that are not suitable for surgery, immunotherapy has attracted much attention in recent years. Nevertheless, the prognosis for patients with advanced HCC is not satisfactory. Early detection and diagnosis of HCC are crucial [8, 15]. The search for efficient biomarkers, risk assessment, and the construction of independent risk factor-related prognostic model are indispensable in HCC diagnosis and prognostic prediction. With the emergence of various novel RCDs, cell death has received widespread attention in tumor progression and prognosis assessment. According to recent reports, proptosis, apoptosis and ferroptosis can jointly participate in the regulation of tumor immune microenvironment and predict prognosis of HCC [16, 17]. Additionally, autophagy has been linked to the emergence and progression of several malignant cancers, such as head and neck squamous cell carcinoma, hepatocellular carcinoma, colorectal cancer and breast cancer [18–21]. It can be seen that various types of cell death are crucial in cancer treatment and prevention. Precisely inducing the death of cancer cells is a major challenge for scientific researchers in recent years.

As a new mode of cell death, disulfidptosis cannot be elucidate by aforementioned cell death means [12]. Many studies have indicated that classifying HCC based on different features can also reveal clinical relevance and prognostic assessment [22, 23]. Aim to investigate the clinical relevance of HCC subtypes based on DRGs features, we collected DRGs from previously published articles and extracted co-expressed genes with DEGs in HCC. Two clusters (C1 and C2) were generated by k-mean cluster analysis of the 45 co-expressed genes. Notably, we found that cluster C1 indicated unfavorable prognosis. The results of GO and KEGG functional enrichment indicate that the high-risk group is primarily concentrated in the regulation of cell division and the cell cycle. To further identify DRGs associated with prognosis, we adopted LASSO Cox analysis, univariate Cox regression analysis and multivariate Cox regression analysis to screen out three hub genes (SPP2, CDCA8 and RDH16). In addition to its association with HCC prognosis, SPP2 is related to colorectal cancer, liver cancer, leukemia and pancreatic cancer [24–27]. RDH16 is also associated with the prognosis of breast cancer and cholangiocarcinoma [28, 29]. CDCA8, functions as a cell cycle regulatory gene, has been reported to participate in the occurrence and development of various cancers such as thyroid and prostate cancer, liver cancer, ovarian cancer and bladder cancer [30–33]. Based on the above hub genes, we calculated the risk score for each HCC sample and divided the LIHC training set into two distinct risk subgroups. Moreover, we compared tumor immune, somatic mutations, and clinical features between the two subtypes. The results showed that the high risk score subgroup had higher somatic mutation frequency, higher immune score, and worse clinical prognosis. These results suggest that disulfidptosis features can be used as independent predictors of HCC prognosis like other RCD. Moreover, compared to traditional clinical staging based on clinical characteristics, the disulfidptosis risk score feature demonstrates greater accuracy in prognostic prediction.

In terms of immune infiltration, HCC represents a typical example of the relationship between the tumor microenvironment (TME) and tumor development, the risk score was found to be positively correlated with immune cell infiltration [34]. The immune microenvironment of HCC is a mixture of liver cancer cells, stromal cells, cytokines and various proteins, which together contribute to the high incidence of HCC metastasis. TAMs and Tregs in TME promote tumor development and metastasis [35]. Tregs represent the predominant population of suppressive cells within the TME, and their presence is intricately linked to the progression, invasiveness, and metastasis of tumors. Tregs are distinguished by the expression of the transcription factor Foxp3, and their functional repertoire encompasses a spectrum of mechanisms, encompassing cell–cell interactions and the release of inhibitory molecules [36]. In our study, we assessed the enrichment level of two subgroups of immune cells and observed a positive correlation between risk scores and immune infiltration. Anti-CTLA-4 disruption leads to cross-talk between Foxp3 Tregs and antigen-presenting cells, thereby promoting autoimmune responses. Coincidentally, we found that the high-risk group exhibited higher CTLA-4 expression and immune score (Tregs) compared to the low-risk group. These results align with the perspectives presented by Alissafi et al. [37]. In a recent Phase III randomized controlled trial (RCTs), the combination of tyrokinase inhibitors (TKIs) and ICI appears to be promising for the treatment of advanced HCC. Combination of TKIs with single-dose ICIs reduces immune-related adverse events (irAEs), which is particularly attractive in high-risk populations [38]. In our study, it is found that the expression of ICI-related genes in the high-risk group is significantly higher than that in the low-risk subtype, which means that the high-risk group had better therapeutic effect on ICI combined with immunomodulators. In line with the previous results, the TIDE database similarly indicates a diminished response to immunotherapy within the high risk score group. This suggests that the emerging ICI/TKI combination therapy for HCC appears to be a rational approach. In term of systemic therapy, the oral multi-tyrosine kinase inhibitor sorafenib currently stands as the sole evidence-based therapy for advanced HCC in patients with intact liver function [39]. It is important to note that the due to toxic or tumor progression and stop using sorafenib in HCC patients, capecitabine may be an effective and safe second-line treatment. In the assessment of drug sensitivity in the two risk subgroups, we did not observe significant differences in sensitivity to sorafenib. However, in the assessment of sensitivity to gemcitabine, the high-risk group exhibited significantly higher sensitivity compared to the low-risk group. This suggests that gemcitabine might be an effective second-line treatment when sorafenib resistance occurs. In other prognostic models of cell death, Li et al. similarly found that gemcitabine showed high sensitivity in HCC with high necroptosis-related risk score and suggested adverse clinical outcomes [40, 41]. Despite this, more evidence awaits further validation through clinical trials.

Although ICI combined with conventional cytotoxic drugs or radiotherapy has shown promising results in hepatobiliary and pancreatic system malignancies, in addition to understanding combination therapy, it is important to consider customized therapy with molecular/immune subtypes [35]. Independent of PD-1, PD-L1, and CTLA4 expression, tumor mutation load (TMB) has been shown in recent years to be a valuable biomarker for predicting the response of PD-1/PD-L1 and CTLA4/B7-1 axis inhibitors in cancer patients treated with ICI. TMB is defined as somatic gene coding errors and the total number of base substitutions, gene insertion, or deletion errors detected per million bases (MB) [42]. The increase of mutation burden of certain malignant tumors enhances the immunogenicity of tumor, which leads to the weakening of its immune escape ability. Tumors with higher TMB are more responsive to ICIs therapy and can be applied to many metastatic malignancies, especially cancers caused by strong mutagens and carcinogens (e.g., non-small cell lung cancer, small cell lung cancer and melanomas) and colorectal cancer with high microsatellite instability [43]. However, TMB is present at lower levels in HCC than in other solid tumors, and its predictive value remains controversial and has not been fully demonstrated. There currently seems to be a consensus that high TMB is associated with reduced survival [44]. TTN was considered to be the most frequently mutated gene in the pan-cancer cohort, with the highest correlation between the number of mutations and TMB [45]. TP53 gene mutation is closely related to tumor immunity and can be used as an effective biomarker to predict the response of different types of cancer to immunotherapy [46]. In our study, TTN in the high-risk group was higher than that in the low-risk group, which is consistent with the fact that the high-risk group predicted poor outcomes. From the perspective of TME, Xie et al. suggested that TMB was positively correlated with Thelper (Th) 2, Th17, and gamma-delta T (Tgd) cell infiltration [47]. In another study, Gao et al. showed that high infiltration of M0 and M2 macrophages, naive CD4 + T cells, and low infiltration of CD8 + T cells were associated with poor prognosis, which is consistent with our findings [48].

DNA hypermethylation is closely associated with the initiation of cancer. Specifically, some copies of tumor suppressor genes undergo hypermethylation or natural mutations [49]. In order to fully understand the correlation between the methylation level of hub gene and clinical features, we analyzed the expression level of hub gene under different clinical features. The results showed that hypermethylation was associated with poor clinical prognosis in HCC, which was consistent with previous conclusions [46]. To verify the protein expression of hub gene in HCC tissues, we used the HPA database to perform immunohistochemical visualization analysis of hub genes. The results suggest that the expression of CDCA8 is upregulated in HCC tissues while the expression of RDH16 is downregulated in HCC tissues. Unfortunately, we did not find data related to SPP2, which may be the reason why SPP2, as a secreted protein, is too low in HCC tissues.

Although a prognostic model based on the disulfidptosis risk score showed good accuracy, our study still had some limitations. First of all, the sample of this study is limited, and further cohort studies are needed to verify the risk assessment model. Secondly, we conducted a complete bioinformatics study and did not further study the mechanism of the target genes on HCC, so it is necessary for future researchers to carry out basic experiments to further explore the mechanism. Additionally, clinical data related to viral infection between different subgroups are lacking in our data, so bias in prognostic assessment may occur as the etiology of underlying liver disease might have a prognostic impact. Of note, most of the 2774 DRGs we selected from the results of CRISPR-Cas9 screen, so the specific relationship between hub gene and disulfidptosis needs to be confirmed by further experiments.

Taken together, our study proposed a DRGs clustering method and developed a risk assessment model for predicting prognosis and response to immunotherapy and chemotherapy in HCC, which is beneficial to guiding the clinical treatment strategies.

## Conclusions

HCC patients were classified into subgroups by unsupervised clustering based on different disulfidptosis features. We discovered that patients with high risk score had weaker response to immunotherapy and were associated with an adverse prognosis of HCC, suggesting that HCC patients with certain disulfidptosis characteristics may benefit from immunotherapy. In addition, gemcitabine maybe an effective therapeutic agent for the treatment of HCC in the high risk score subgroup.

### Supplementary Information

**Additional file 1: Fig. S1.** CIBERSORT immune scores of 22 kinds of immune cells in different subgroups. **Fig. S2.** Immunohistochemical and total protein expression results of CDCA8 and RDH16. **Fig. S3.** CDCA8 methylation expression under different clinical characteristics. **Fig. S4.** SPP2 methylation expression under different clinical characteristics. **Fig. S5.** RDH16 methylation expression under different clinical characteristics

**Additional file 2: **DRGs list and Clinical Baseline Data from TCGA,GEO, and ICGC

**Additional file 3: **GO and KEGG Analysis Results

**Additional file 4: **GSEA Analysis Results

## Data Availability

Transcriptome data, clinical information and somatic mutations data of the study were collected from TCGA database (https://portal.gdc.cancer.gov/). The verification set data are downloaded from GEO database (https://www.ncbi.nlm.nih.gov.) and ICGC database (https://dcc.icgc.org/). Methylation data and total protein in UALCAN database (http://ualcan.path.uab.edu/index.html.). Immunohistochemical data were obtained from The Human Protein Atlas (HPA) database (http:// www.proteinatlas.org). Due to the fact that these are public databases, all patients should have been told and given their consent prior to the release of their information.
