# Supplementary material for: Disulfidptosis-related signatures for prognostic and immunotherapy reactivity evaluation in hepatocellular carcinoma
Source: Eur J Med Res. 2023 Dec 6;28:571. doi: 10.1186/s40001-023-01535-3 (PMC10698993; doi:10.1186/s40001-023-01535-3)
Supplement: Supplementary file 1 — Additional file 1: Fig. S1. CIBERSORT immune scores of 22 kinds of immune cells in different subgroups. Fig. S2. Immunohistochemical and total protein expression results of CDCA8 and RDH16. Fig. S3. CDCA8 methylation expression under different clinical characteristics. Fig. S4. SPP2 methylation expression under different clinical characteristics. Fig. S5. RDH16 methylation expression under different clinical characteristics [file 40001_2023_1535_MOESM1_ESM.pdf]

Additional file 1

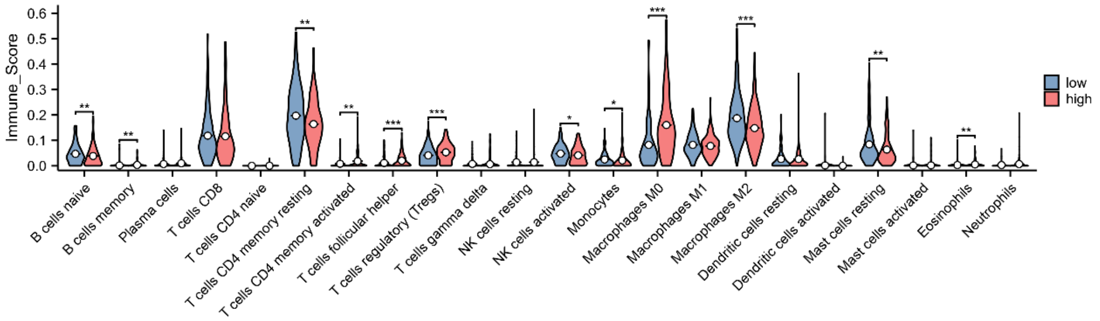

Fig. S1 CIBERSORT immune scores of 22 kinds of immune cells in different subgroups

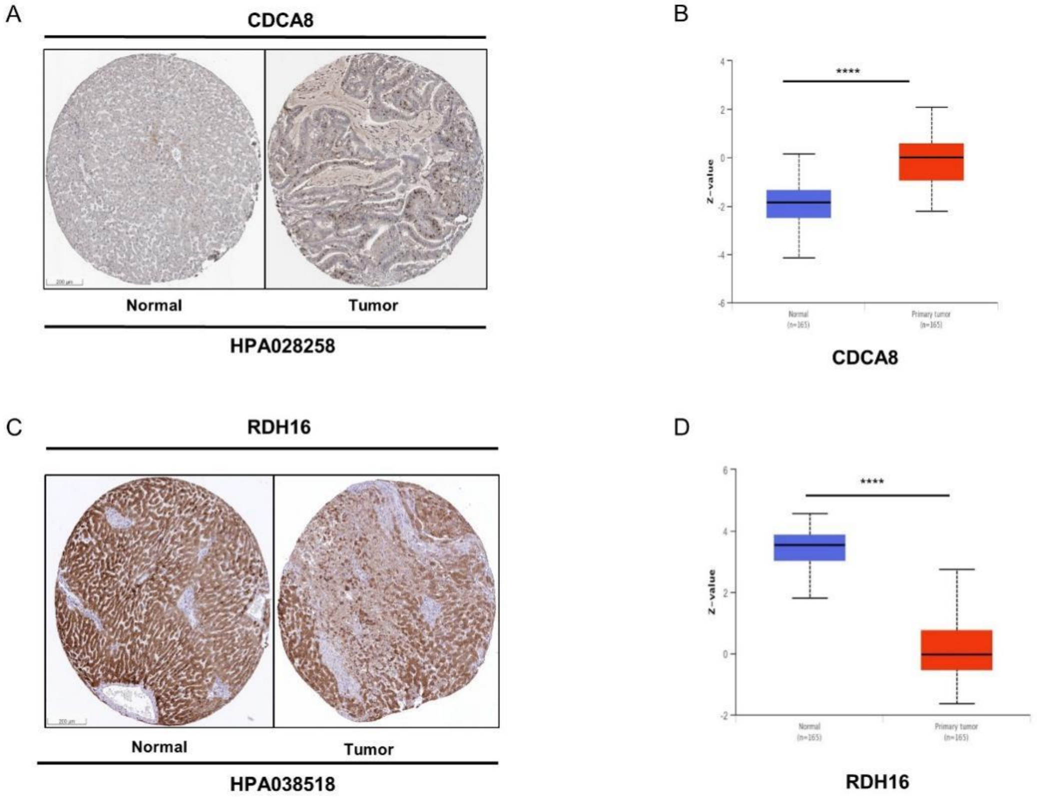

Fig. S2 Immunohistochemical and total protein expression results of CDCA8 and RDH16. (A, C) Immunohistochemical results of HCC tiusse and normal tissue with CDCA8 and RDH16. (B, D) Total protein expression levels of CDCA8 and RDH16 in HCC tissues.

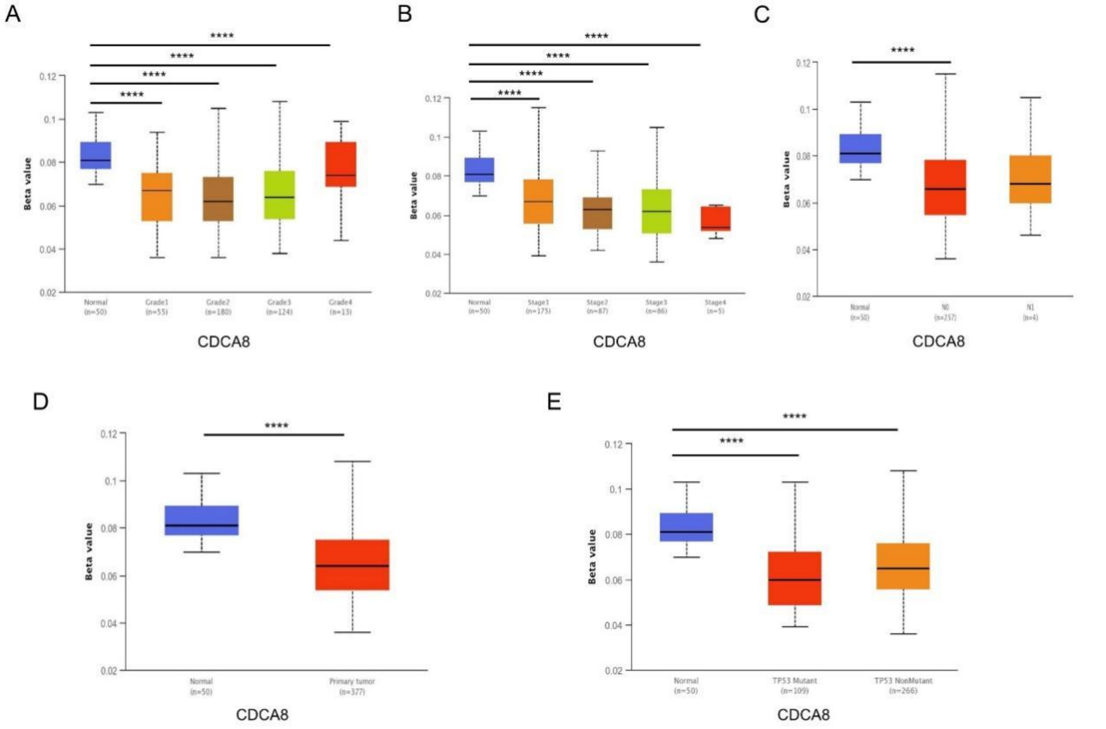

Fig. S3 CDCA8 methylation expression under different clinical characteristics(A-C) Methylation levels of CDCA8 under different TMN characteristics. (D) Methylation levels of CDCA8 in different disease states. (E) Methylation levels of CDCA8 between different TP53 mutation subgroups.

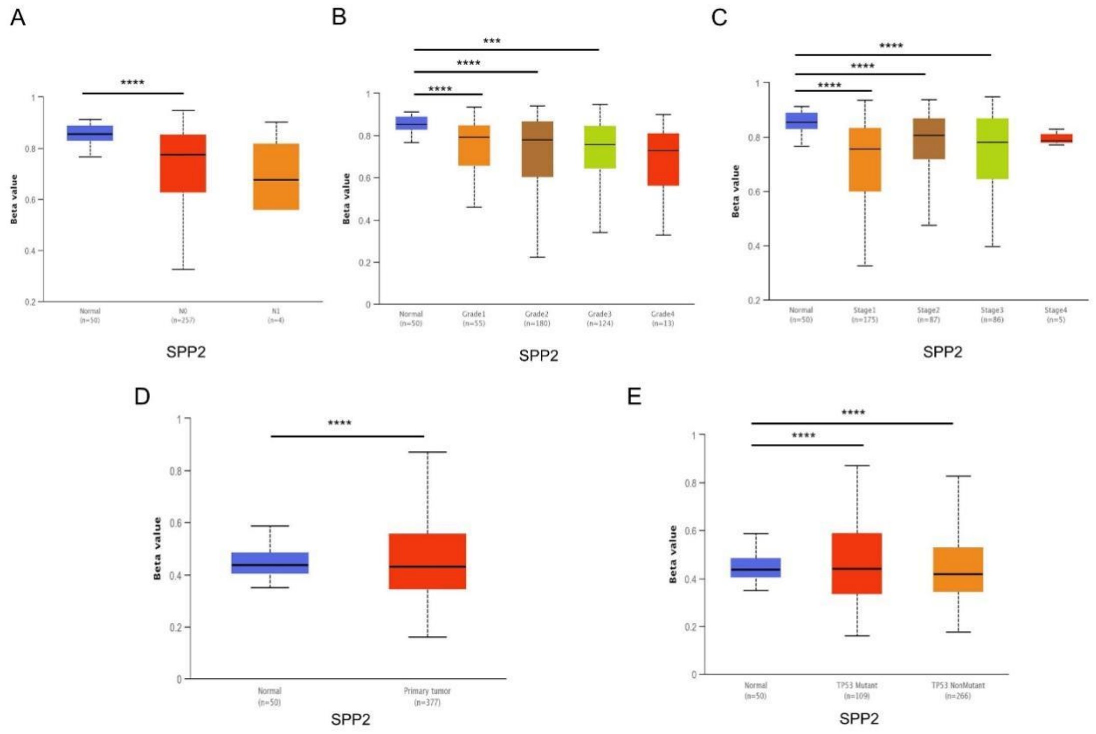

Fig. S4 SPP2 methylation expression under different clinical characteristics (A-C) Methylation levels of SPP2 under different TMN characteristics. (D) Methylation levels of SPP2 in different disease states. (E) Methylation levels of SPP2 between different TP53 mutation subgroups.

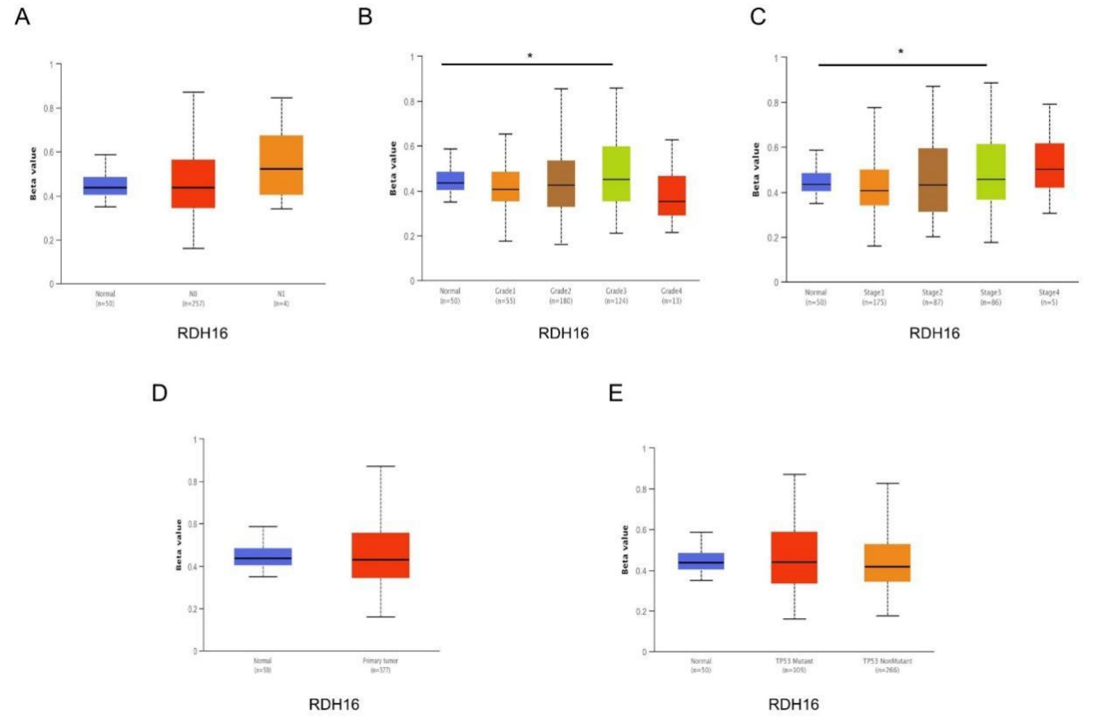

Fig. S5 RDH16 methylation expression under different clinical characteristics (A-C) Methylation levels of RDH16 under different TMN characteristics. (D) Methylation levels of RDH16 in different disease states. (E) Methylation levels of RDH16 between different TP53 mutation subgroups.
